# Room-Temperature Metal-Catalyzed Hydrogen Borrowing Alkylation

**DOI:** 10.1021/acscatal.5c07305

**Published:** 2026-01-10

**Authors:** Elliot P. Bailey, Timothy J. Donohoe, Martin D. Smith

**Affiliations:** Chemistry Research Laboratory, 6396 University of Oxford, 12 Mansfield Road, Oxford OX1 3TA, U.K.

**Keywords:** alcohols as alkylating agents, carbon−carbon bond formation, carbon−nitrogen bond formation, functional group tolerance, enantioselectivity, regioselectivity, sustainability, green chemistry

## Abstract

Hydrogen borrowing describes a one-pot multistep sequence in which an alcohol is used as an alkylating agent. In comparison to a traditional alkylation reaction using alkyl halides, this is an attractive strategy: alcohol substrates are commercially abundant and stable, the process uses catalytic amounts of metal and base, and water is generated as the sole byproduct. Since seminal reports in the early 2000s, the field has been investigated extensively, but most hydrogen borrowing reactions operate under a high-temperature regime (76–200 °C), particularly those involving carbon–carbon bond formation. This review provides an overview of the current state of the art in room-temperature (≤30 °C) hydrogen borrowing reactions, including both carbon–carbon and carbon–nitrogen bond formation.

## Graphical abstract

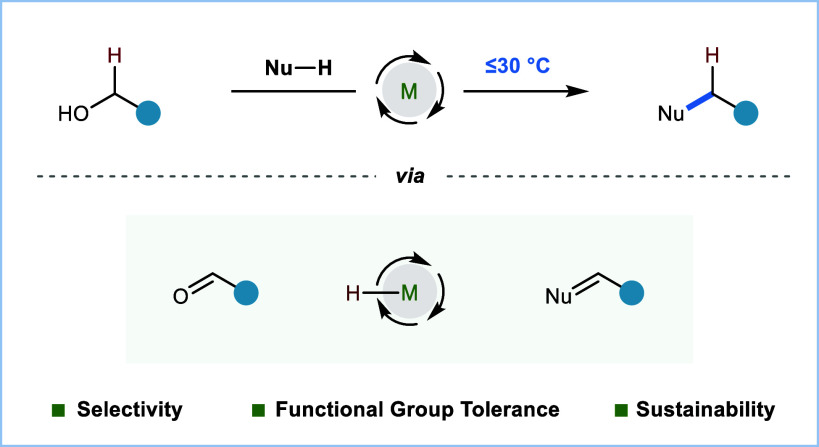

## 1 Introduction to Transition-Metal-Catalyzed Hydrogen Borrowing Alkylation

Hydrogen borrowing describes a one-pot multistep sequence in which an alcohol is used as an alkylating agent. A transition-metal (and base)-catalyzed hydrogen borrowing alkylation of a ketone is outlined as an exemplar process (Figure 1a). Base-mediated ligand exchange between a weakly bound ligand at a transition metal complex **1** and an alcohol **2** leads to the formation of a metal alkoxide complex **3**. This species undergoes β-hydride elimination to generate an aldehyde **4** (or ketone if a secondary alcohol is used) as well as a metal hydride species **5**. A base-catalyzed aldol reaction with ketone **6** then delivers enone **7**, which is poised for conjugate reduction by the *in situ* generated metal hydride **5**. This regenerates the alkylated product **8** and a metal complex with a vacant coordination site, which allows another equivalent of alkoxide to ligate and propagate the reaction.^1,2^ This approach offers many benefits over traditional alkylation with alkyl halides, which are toxic and generally unstable to a number of processes, including solvolysis, elimination, and radical formation (in light). These reactions often require a stoichiometric base, which leads to the generation of stoichiometric waste. In contrast, the hydrogen borrowing approach offers a number of benefits: (i) alcohols are commercially abundant, stable, and safe to handle; (ii) in principle, the reactions can be catalytic in base and water is the sole by product of the reaction; (iii) this approach tolerates secondary alcohols, expanding the scope of pro-electrophile coupling partners; (iv) the use of a catalytic transition metal mimics the dynamics of dropwise addition of a reactive species (e.g., an aldehyde) in an alkylation reaction, but in an operationally friendly one-pot protocol. In other words, a metal hydride species and an aldehyde are generated after the oxidation step, and this hydride must be consumed (via reduction of an enone) in order to allow another alcohol oxidation step to occur (via generation of a vacant coordination site).

**Figure 1:**
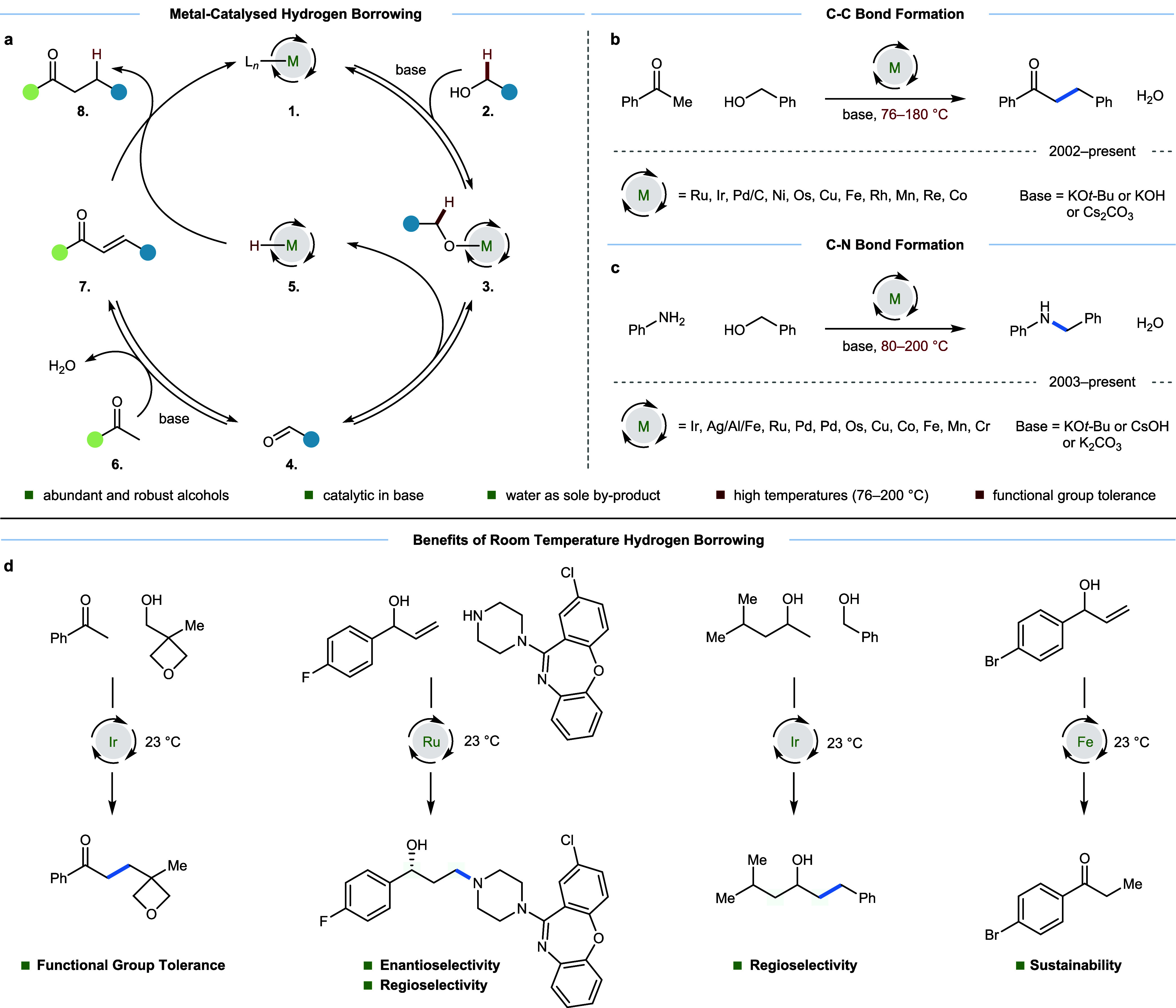
Introduction to hydrogen borrowing alkylation. (a) Transition-metal- and base-catalyzed hydrogen borrowing enolate alkylation mechanism. (b) Summary of conditions used in hydrogen borrowing alkylation of acetophenone with benzyl alcohol, revealing the common theme of use of high temperatures (76–180 °C). (c) Summary of conditions used in hydrogen borrowing alkylation of aniline with benzyl alcohol, revealing the common theme of use of high temperatures (80–200 °C). (d) Benefits of room-temperature hydrogen borrowing including functional group tolerance, enantioselectivity, regioselectivity, and sustainability.

## 2 Hydrogen Borrowing Typically Requires High Temperatures (76–200 °C)

Over the last two decades, the field has been investigated extensively and a panoply of (pro-nucleophile) substrates have been demonstrated in hydrogen borrowing reactions. Alongside a large number of diverse substrates, numerous reaction conditions, and transition metal catalysts that can enable hydrogen borrowing have been reported. The alkylation of acetophenone (Figure 1b) or aniline (Figure 1c) with benzyl alcohol is outlined to illustrate these reports, taking each as exemplary substrates for C–C and C–N bond formation, respectively. These reactions have been reported to be effective with many different metal catalysts, but across the numerous conditions that enable these processes, there is a common theme: hydrogen borrowing alkylation requires high operating temperatures (76–200 °C). High temperatures, in combination with commonly used bases such as hydroxide or *tert*-butoxide, lead to relatively harsh reaction conditions, which we suggest is a reason that the substrates employed in hydrogen borrowing reactions are rarely functional group diverse.^1,3–5^ Complex molecules, sensitive or reactive functional groups, nitrogen-containing heterocycles, and strained rings are rarely exemplified as successful substrates.

In principle, the realization of hydrogen borrowing alkylation at lower temperatures could overcome these limitations and would therefore be of appreciable benefit to the academic and industrial communities (Figure 1d). To aid in this endeavor, this review highlights room-temperature (≤30 °C) metal-catalyzed hydrogen borrowing reactions for both carbon–carbon and carbon–nitrogen bond formation. Hydrogen borrowing via biocatalysis, often performed between ambient and physiological temperature, is omitted as this subfield has been recently and excellently reviewed.^6^

## 3 Room-Temperature C–C Bond-Forming Hydrogen Borrowing Reactions

In 2013, Quintard, Rodriguez, and co-workers reported an enantioselective alkylation of ketoesters **9** with allylic alcohols **10** via a bicatalytic iron and iminium strategy (Figure 2a).^7^ Knölker complex precatalyst **11** is converted to an active form by reaction with Me_3_NO, which liberates CO_2_ and trimethylamine, creating a necessary vacant coordination site at the iron center. This iron species is capable of oxidation of allylic alcohol **10** to generate an iron hydride and an α,β-unsaturated aldehyde, which is poised for condensation with chiral aminocatalyst **12** to form an α,β-unsaturated imminium. Rapid conjugate addition by the enolate derived from ketoester **9** and subsequent hydrolysis and reduction of the aldehyde by the iron hydride liberates the alkylated product **13**, which is in equilibrium with the corresponding hemiacetal **14**. There are two similar sets of conditions disclosed, each varying the amounts of reaction components slightly at either room temperature or 10 °C (Figure 2a). The reaction was successful with both cyclic and acyclic substrates, generating products with good e.r., and moderate to good yields over extended reaction times (Figure 2a, **15**: 42%, 21 h, 75:25 d.r., 92:9 e.r.; **16**: 64%, 69 h, 9:1 d.r., 91.5:8.5 e.r.; **17**: 63%, 40 h, 9:1 d.r., 93:7 e.r.). Before this publication, the Knölker complex **11** had been used in both alcohol oxidation at elevated temperatures (60 °C),^8^ and hydrogen transfer processes at room,^9,10^ and elevated temperatures (80–100 °C),^11–14^ but this was the first time it had been applied to a hydrogen borrowing process. To catalyze hydrogen borrowing at low temperatures with an earth-abundant metal is particularly notable, and this reaction also demonstrates alternative pathways for nucleophiles to attack *in situ* generated aldehydes (in a Michael addition reaction in this case).

**Figure 2:**
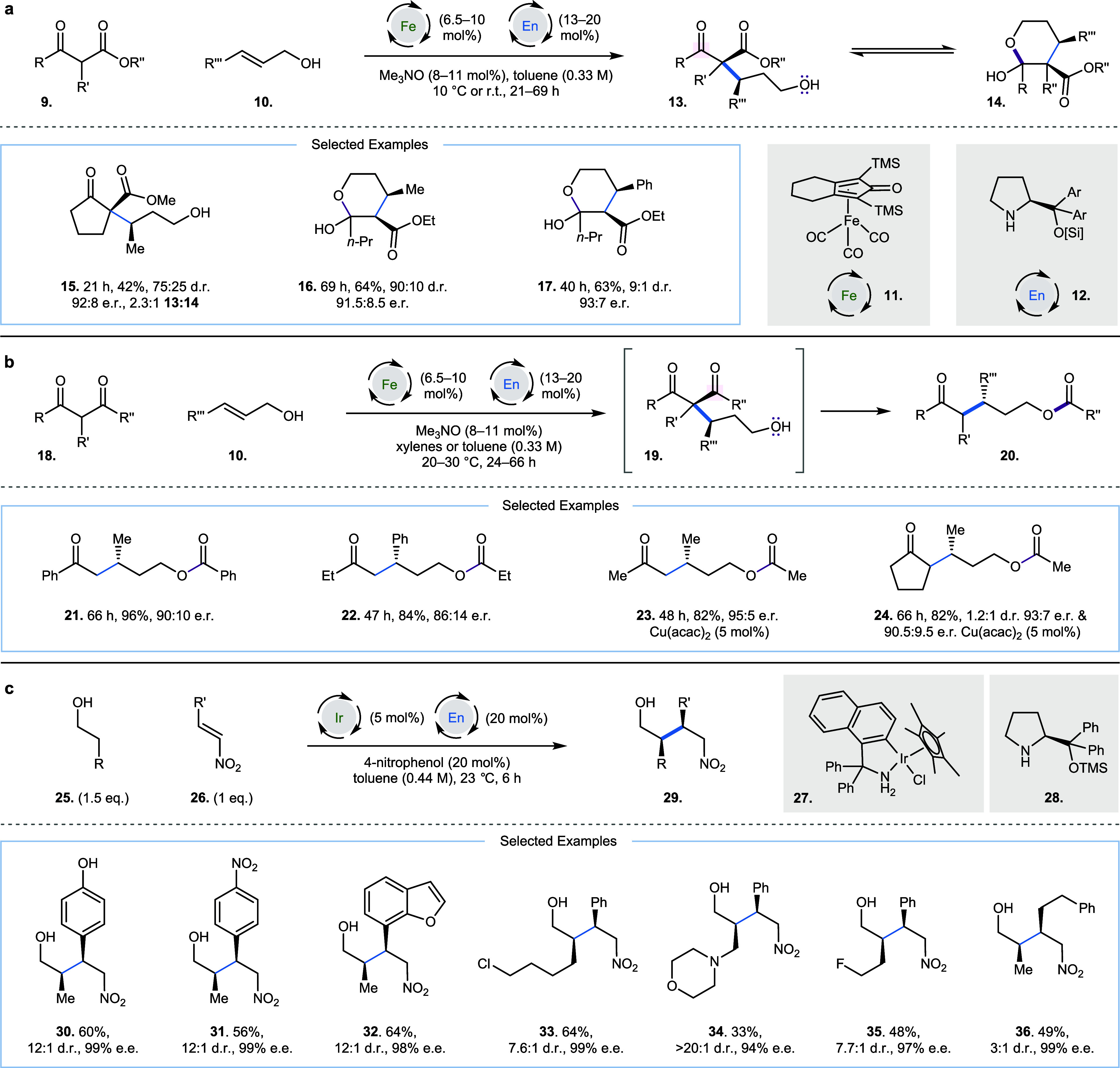
Organocatalytic room-temperature enantioselective alkylation of alcohols. (a) Alkylation of allylic alcohols with ketoesters at 10 °C or room temperature, where Ar = Ph and [Si] = TMS. (b) Alkylation of allylic alcohols with diketones at room temperature, Ar = Ph, [Si] = TMS; Ar = 3,5-bis­(CF_3_)­C_6_H_3_, [Si] = TMS; Ar = Ph, [Si] = TBDMS. (c) Alkylation of alcohols via an enamine reaction with nitroalkenes.

Further publications in 2014 ^15^ and 2016 ^16^ expanded the scope of this reaction to employ diketones **18** in similar transformations with enhanced enantioselectivity. The alcohol group in the product **19** spontaneously cyclizes to generate ketoester products **20** (Figure 2b, **21**: 96%, 66 h, 90:10 e.r.; **22**: 84%, 47 h, 86:14 e.r.). The authors also describe the beneficial effect of Cu­(acac)_2_ (5–15 mol %) on enantioselectivity; it is believed to aid the turnover-limiting Michael addition to allow better differentiation of two diastereomeric transition states (Figure 2b, **23**: 82%, 48 h, 95:5 e.r.; **24**: 82%, 66 h, 1.2:1 d.r., 93:7 e.r. and 90.5:9.5 e.r. respectively). This work included a functional group tolerance study derived from the work of Glorius and co-workers,^17^ demonstrating tolerance of alkyl chlorides, 1-methylindole, 2-oxindole, disubstituted alkynes, but notably the intolerance of heterocycles such as 1-methylimidazole and pyridine.^16^ In 2018, Quintard and co-workers described a similar alkylation strategy, which enabled the generation of spirolactone products.^18^

In 2025, Zou, Wu, Zhao, and co-workers reported the enantioselective organocatalytic alkylation of alcohols **25** with nitroalkenes **26** (Figure 2c).^19^ Oxidation of an alcohol **25** by a cyclometalated iridium catalyst **27** followed by condensation of the amine catalyst **28** generates an enamine, which can undergo a 1,4-addition reaction with nitroalkenes **26**; hydrolysis and subsequent aldehyde reduction can generate alkylated products **29**. Chiral aldehyde intermediates generated during the reaction do not undergo substantive racemisation; the authors suggest this is a consequence of these intermediates being generated in low concentrations and being rapidly trapped in a reduction process to yield **29** with high diastereoselectivity.^20^ The reaction demonstrated impressive functional group tolerance (**30**–**36**) including phenol, morpholine, alkyl halide, and silyl ether groups.

In 2020, Zhao and co-workers described a highly efficient room temperature Guerbet reaction (Figure 3a), including an enantioselective variant (Figure 3b) between primary and secondary alcohols (**37** and **38**, respectively, to generate **39**).^21^ This required a cyclometalated iridium catalyst **40** (1 mol %), for the racemic reaction (**41**–**45**) and a chiral ruthenium catalyst **48** (1 mol %) for the enantioselective variant (**49**–**52**), in addition to the cocatalyst pentan-3-one (5 mol %) for both reactions. Pentan-3-one initiates the reaction by acting as a hydride acceptor from the iridium hydride generated *in situ* via oxidation of either the primary or secondary alcohol starting material (**37** and **38**, respectively). This allows for the formation of an aldehyde and a ketone derived from secondary and primary alcohol starting materials (**37** and **38**, respectively), which can then undergo a base-catalyzed aldol reaction to generate an enone. This enone is poised for double reduction by iridium hydride species (iridium hydride formation is facilitated by the reduced form of the cocatalyst, 3-pentanol) to ultimately give the alkylated secondary alcohol product (**39** for the racemic reaction and **47** for the enantioselective reaction).

**Figure 3:**
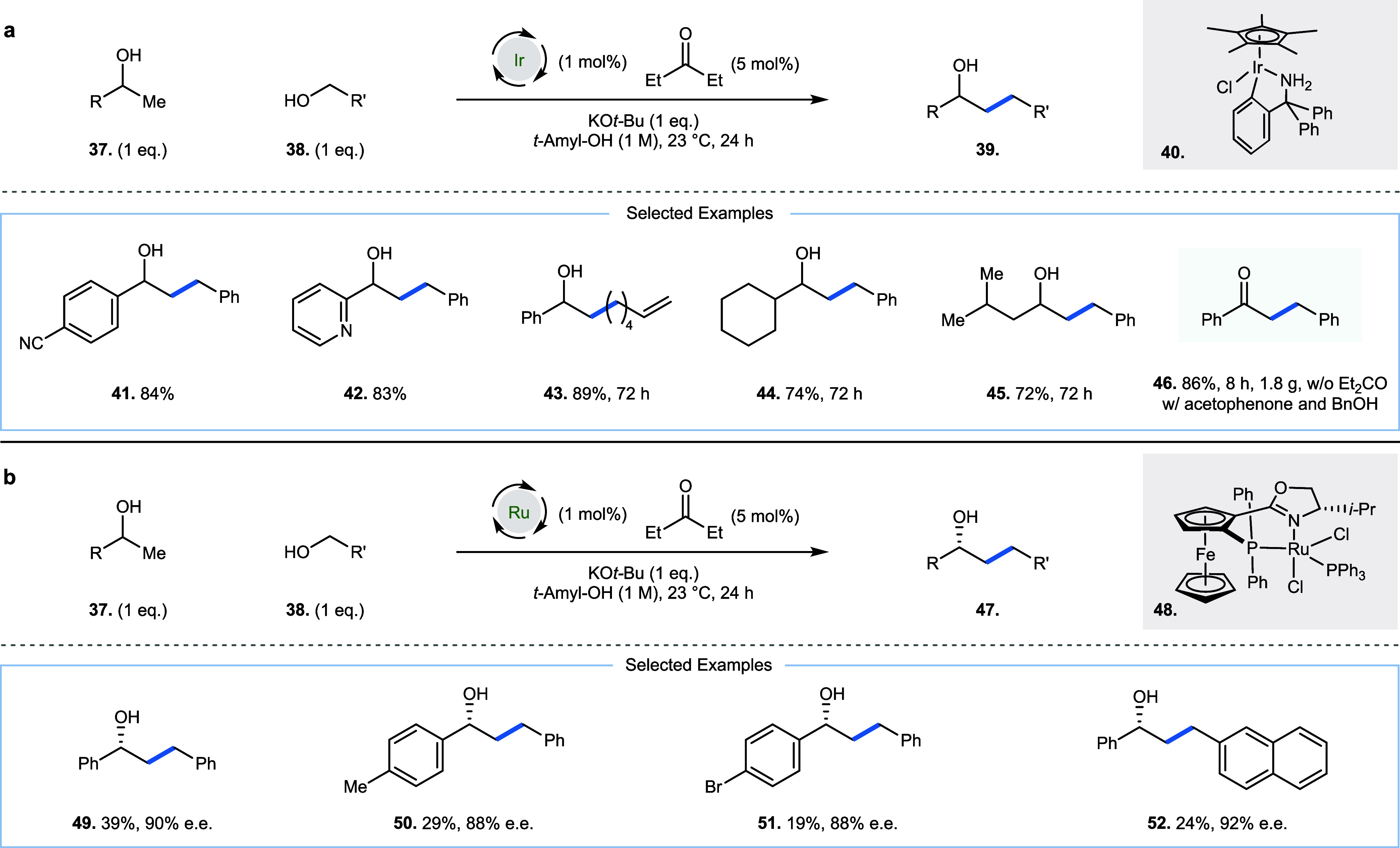
Room-temperature Guerbet reaction between secondary and primary alcohols. (a) Parent racemic reaction. (b) Enantioselective variant.

The alcohol substrates in each reaction broadly demonstrate the following features: one is benzylic and secondary (**37**), while the other is benzylic and primary (**38**, Figure 3a). After oxidation, both a ketone and an aldehyde without enolizable protons will be generated, which leads to a rapid crossed aldol reaction, before double reduction generates products **39** within a reaction time of 24 h (**41, 42**). When a nonbenzylic primary alcohol is used, an enolizable aldehyde is generated and an extended reaction time is required (72 h, **43**); this is also the case when a nonbenzylic secondary alcohol is used (**44**). The use of low temperature to disfavor nonproductive, but reversible, aldol reaction pathways allow the authors to demonstrate regioselective alkylation for a host of substrates (**45**). A room temperature hydrogen borrowing reaction between acetophenone and benzyl alcohol is also described in this publication on a gram scale, achieved by removing the pentan-3-one cocatalyst additive from the original conditions (Figure 3a, 1.8 g **46** obtained). Finally, the authors demonstrate the viability of an enantioselective variant at room temperature with chiral ruthenium complex **48**, which can generate secondary alcohols in good e.e. and modest yield (Figure 3b, **49**–**52**, 88–92% e.e. and 19–40% yield).

In 2022 Wang and co-workers, and Wang and co-workers described two related approaches (Figure 4a,b) to the enantio- and diastereoselective alkylation of allylic alcohols (**53**) with amino acid derivatives (**54** and **62**).^22,23^ Oxidation of an allylic alcohol by ruthenium complexes (**56** and **64**) generates α, β-unsaturated ketones, which can undergo Michael addition with enolates derived from **54** and **62**. Subsequent diastereoselective reduction of the carbonyl delivers products **55** and **63**, respectively. For linear amino acid derivatives (**54**), the authors remark that a low catalyst loading (1–2 mol %) helps to increase the d.r. of the products (**57**–**61**), which was attributed to a reduction in the relative rate of the hydrogen borrowing steps (Figure 4a). Two-phase transfer catalysts (TBAHS and 18-crown-6, each 0.5 equiv) are required, which were found to increase the rate of the Michael addition reaction, increasing both the yield and d.r. of the product. The high d.r. observed was attributed to a dynamic kinetic asymmetric transformation: fast reversible epimerization via deprotonation of the C–H adjacent to the amino acid moiety precedes a fast diastereoselective carbonyl reduction of one of the enantiomers by chiral ruthenium complex **56** to deliver the product. This contrasts with the reaction with cyclic precursors (**62**, Figure 4b), in which the authors demonstrate that the stereogenic center generated after 1,4-addition leads to asymmetric induction at the secondary alcohol stereogenic center (**66**–**70**), formed after reduction by chiral ruthenium complex **64**. For these cyclic precursors, a copper catalyst [Cu­(MeCN)_4_]­BF_4_ and chiral phosphinooxazoline ligand (**65**) was necessary for enantioselective Michael addition of enolates derived from **62**. In 2025, Dong, Wang, and co-workers described a related method for the synthesis of enantiopure δ-hydroxy α-amino acids (**71** and **74**–**78**) bearing two contiguous stereocenters, by alkylation of racemic γ-substituted allylic alcohols **10** with ketoimine ester **54** (Figure 4c).^24^ Achiral ruthenium catalyst **72** effected hydrogen borrowing and a chiral copper catalyst (**73**) enabled diastereoselective Michael addition. A phosphate base (K_3_PO_4_) was key to reactivity: commonly used carbonate bases K_2_CO_3_ and Cs_2_CO_3_ led to significant loss of enantioenrichment (84 and 22% e.e., respectively) in the product, which was attributed to enhanced racemic background reaction. At room temperature, heteroaromatic substrates including pyridine, furan, thiophene, and indole proved compatible.

**Figure 4:**
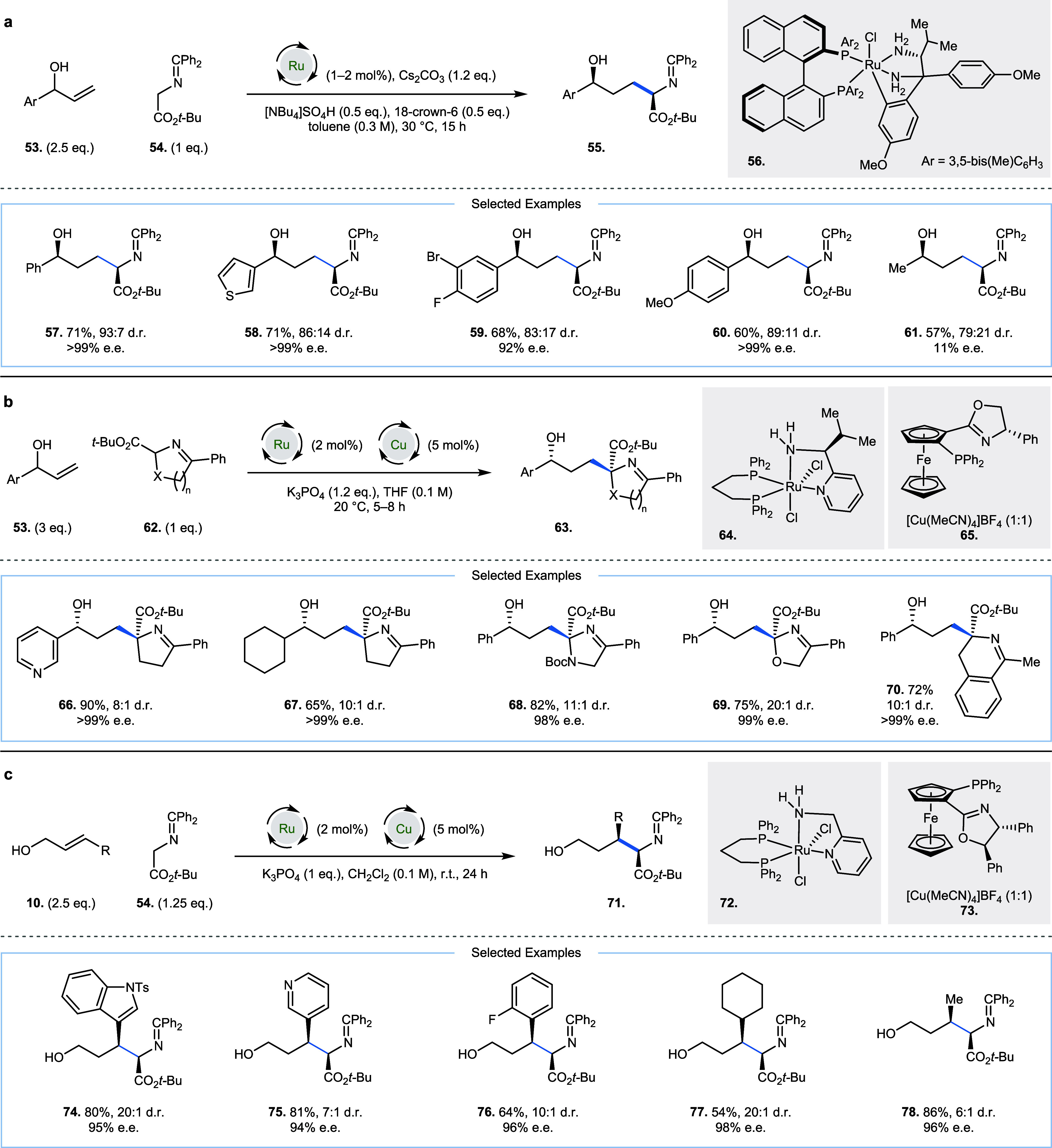
Room-temperature hydrogen borrowing alkylation of allylic alcohols. (a) Enantioselective alkylation with amino acid derivatives, Ar = 3,5-bis­(Me)­C_6_H_3_. (b) Enantioselective alkylation with cyclic nucleophiles. (c) Enantioselective alkylation with amino acid derivatives.

In 2025, Lin, Wang, and co-workers described the enantioselective hydrobenzylation of racemic allylic alcohols **79**, using aryl hydrazones **80** as pro-nucleophiles at 10 °C (Figure 5a).^25^ High enantioselectivity was observed up to 30 °C, but lower temperature, as well as increasing steric bulk (by introduction of a mesityl group) at the site of conjugate addition, led to a significant increase in d.r. of the alcohol products (**81**–**85**). Remarkably, a single ruthenium catalyst **56** enabled this reactivity, which could affect the hydrogen borrowing oxidation and reduction as well as hydrazone activation and conjugate addition reactions.

**Figure 5:**
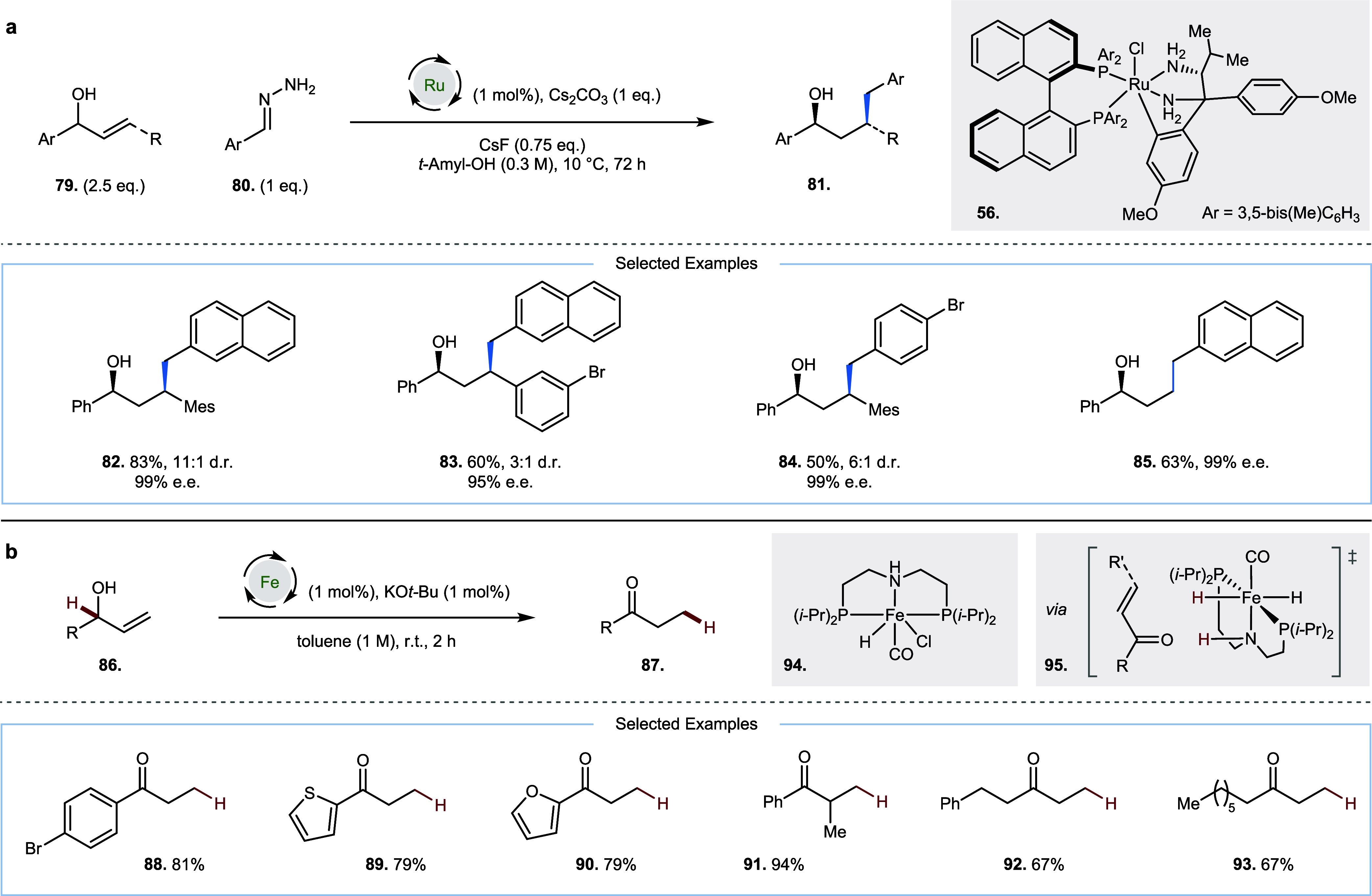
Room-temperature hydrogen borrowing with allylic alcohols. (a) Enantioselective hydrobenzylation with hydrazone pro-nucleophiles, Ar = 3,5-bis­(Me)­C_6_H_3_. (b) Isomerization with an iron catalyst.

In 2018, de Vries and co-workers described a room-temperature iron-catalyzed isomerization of allylic alcohols **86** to ketones **87**, proceeding via a hydrogen borrowing mechanism (Figure 5b, **88**–**93**).^26^ In this case, there is no intermediate reaction between the hydrogen borrowing (oxidation) and hydrogen returning (reduction) steps; however, it is noteworthy that the iron catalyst **94** can perform both the oxidation and reduction steps at room temperature. These catalysts were first trialed at 80 °C for 1 h and later proved to be proficient at room temperature. Density functional theory was used to indicate that a hydrogen borrowing mechanism, involving oxidation of the allylic alcohol **86** and conjugate reduction of the subsequent enone (see **95**) to the ketone product **87** was kinetically viable at room temperature. This was in contrast to an alternative mechanism via hydrometalation of the alkene and subsequent β-hydride elimination, which would generate the same products, but was excluded due to a high overall effective Gibbs free energy barrier compared to dehydrogenation of the allylic alcohol (217.8 vs 92.1 kJ mol^–1^, respectively).

Visible light-driven hydrogen borrowing reactions, enabling the deligation of carbon monoxide from the iron center in a Knölker precatalyst (**96**) have also been reported (Figure 6). In 2022, Poater, Renaud, and co-workers detailed the alkylation of methyl ketones (**97**) with primary benzylic alcohols; unactivated primary alcohols were also viable substrates under longer reaction times of 16–72 h (Figure 6a, **98**–**103**).^27^ In 2024, the group expanded this work to use allylic alcohols, delivering γ,δ-unsaturated ketones (as a mixture of *E*/*Z* isomers) and saturated ketones (isolated as inseparable mixtures with the former), while also detailing the use of propargylic alcohols, which delivered alkylated ketone products without alkyne reduction or isomerization (Figure 6a, **103**).^28^ In 2023, Ling, Zhong, and co-workers reported the alkylation of 2-aryl acetonitriles (**104**) with a range of primary alcohols (to generate **105**) after 12 h (Figure 6b, **105**–**107**).^29^ When using secondary alcohols, the corresponding unsaturated product was also observed in good yield (**108**); this is consistent with H_2_ release (from *in situ* generated iron hydride) becoming competitive with the reduction of the corresponding unsaturated product, when 2-aryl acetonitriles are used as pro-nucleophiles (**108**). In 2024, the group expanded this work to demonstrate the alkylation of 2-oxindoles (**109**, to generate **110**) with a range of both primary and secondary alcohols (Figure 6b, **111**–**113**), which required addition of triphenylphosphine as a ligand (5 mol %: 90% yield of product observed with PPh_3_; 55% yield without PPh_3_ in the alkylation of 1-phenylindolin-2-one with benzyl alcohol).^30^ Concurrent to reports by Poater, Renaud, and co-workers in 2022, Sundararaju and co-workers also reported similar reactivity of a related Knölker complex.^31^ The authors demonstrated that ketones such as propiophenone, 1,3-diphenylpropan-1-one, and 1,2-diphenylethan-1-one could undergo methylation with methanol in the presence of visible light. During the course of the reaction, the highest reaction temperature measured was 42 °C, highlighting the intrinsic coupling of heat and light energy when employing visible light: in this case a significant increase in yield of alkylated products was observed with visible light (at 42 °C) compared to running the reaction at 50 °C without visible light (93 and 18% yield, respectively, in an exemplar reaction). These reports highlight how photochemistry can be used to enable ligand dissociation from inactive precatalysts to generate active catalysts capable of effecting hydrogen borrowing reactions at room temperature.

**Figure 6:**
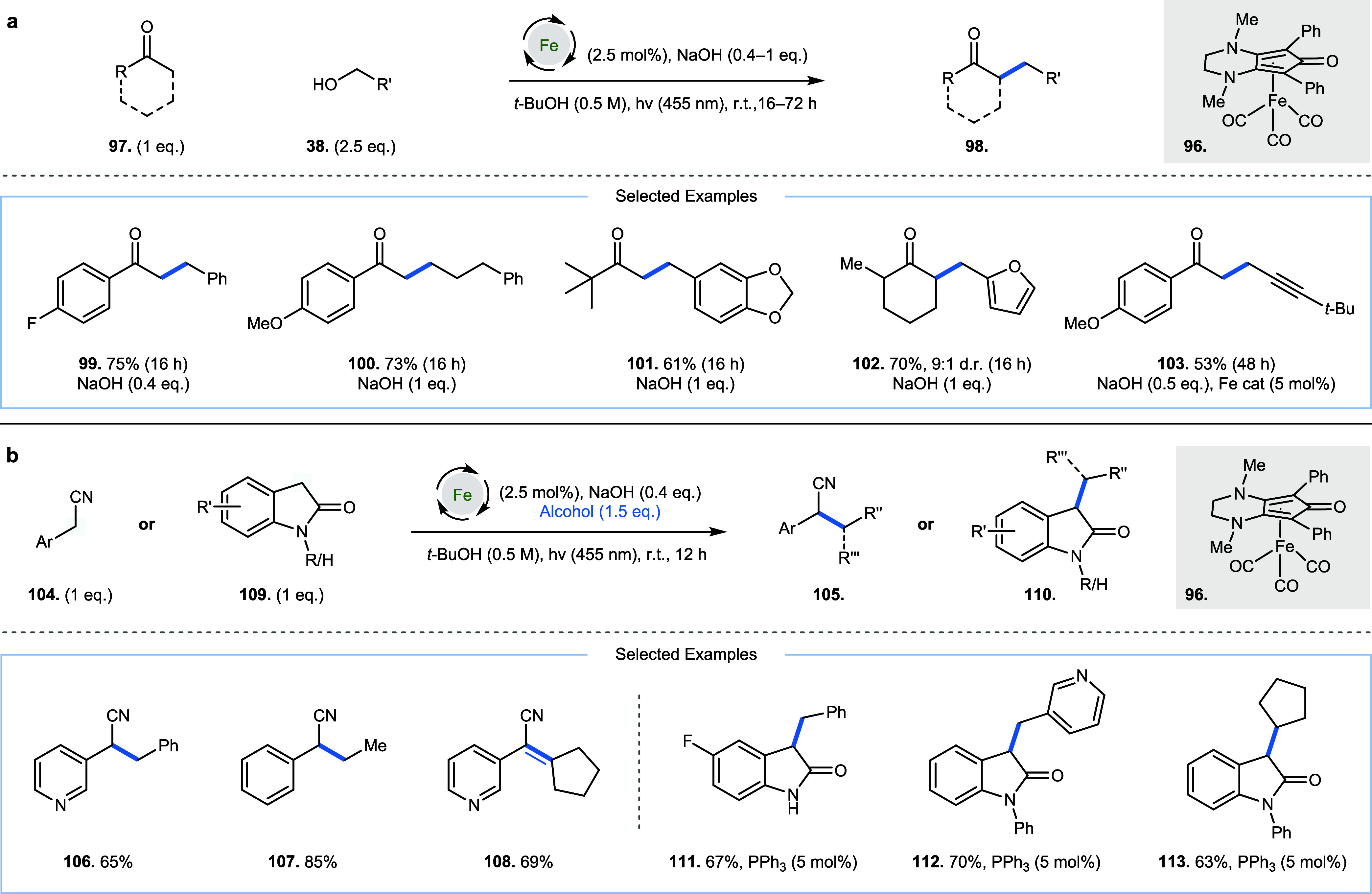
Visible light-driven room-temperature hydrogen borrowing alkylation via carbon monoxide ligand loss from an iron catalyst. (a) Alkylation of ketones with primary alcohols. (b) Alkylation of 2-oxindoles and acetonitriles with primary and secondary alcohols.

In 2024, we described the room temperature hydrogen borrowing alkylation of methyl ketones **114** with primary alcohols **38** to yield products **115** by employing commercially available iridium catalyst **116** (Figure 7a).^32^ The substrates exemplified included functional groups such as pyridine (**117**), oxetane (**118**, **119**), bicyclopentane (**120**, **121**), pyrrolidine (**120**, **121**), piperidine (**122**, **123**), indole (**122**, **123**), alkyl chloride (**124**), alkyl bromide (**125**), silyl ethers (**126**), morpholine (**127**), imidazole (**128**), and (difluoro)­cyclopropanes (**129**). Initially, the work employed a privileged pentamethylphenyl (Ph*) methyl ketone to investigate reactions with diverse alcohols; this substrate disfavors unproductive side reactions at the carbonyl group (such as reduction or aldol reaction) due to steric shielding of the C=O by the arene methyl groups. The Ph* group is cleavable to the corresponding carboxylic acid group by exposure to 2 M HCl in HFIP (1,1,1,3,3,3-hexafluoroisopropanol) at 40 °C for 16 h (derivatization of **119, 121, 124, 125, 127**, and **128**, 70–96% yield). Later, the work was expanded to include aromatic ketones such as acetophenone and 2-, 3-, and 4-methoxy acetophenone. Notably, alcohols possessing base-sensitive functional groups were now tolerated in the reaction at room temperature, which otherwise performed poorly at higher temperatures. This includes functional groups such as oxetane (**119**, 23 and 85 °C; 94% vs 51%), bicyclopentane (**121**, 23 and 85 °C; 73% vs 25%), indole (**123**, 23 and 85 °C; 87% vs 42%), alkyl chloride (**124**, 23 and 85 °C; 89% vs 24%), alkyl bromide (**125**, 23 and 85 °C; 65% vs 24%), silyl protected alcohols (**126**, 23 and 85 °C; 94% vs 26%) and difluorocyclopropanes (**129**, 23 and 85 °C; 59% vs 14%). At room temperature, the reaction was shown to *require* anaerobic conditions to operate; we suggest this is due to the reaction of observed iridium hydride (**130**) species with O_2_, which has been reported to form iridium hydroperoxide species (**131**), leading to catalyst modification and degradation (Figure 7b).^2^ The limitations of this reaction at room temperature were due to catalyst complexation/sequestration by metal-binding heteroatom-containing substrates, which are suggested to inhibit the oxidation step by preventing effective β-hydride elimination (this requires vacant coordination sites on the metal to operate). However, this work demonstrated that labile or complex functionality can be tolerated in hydrogen borrowing at room temperature.

**Figure 7:**
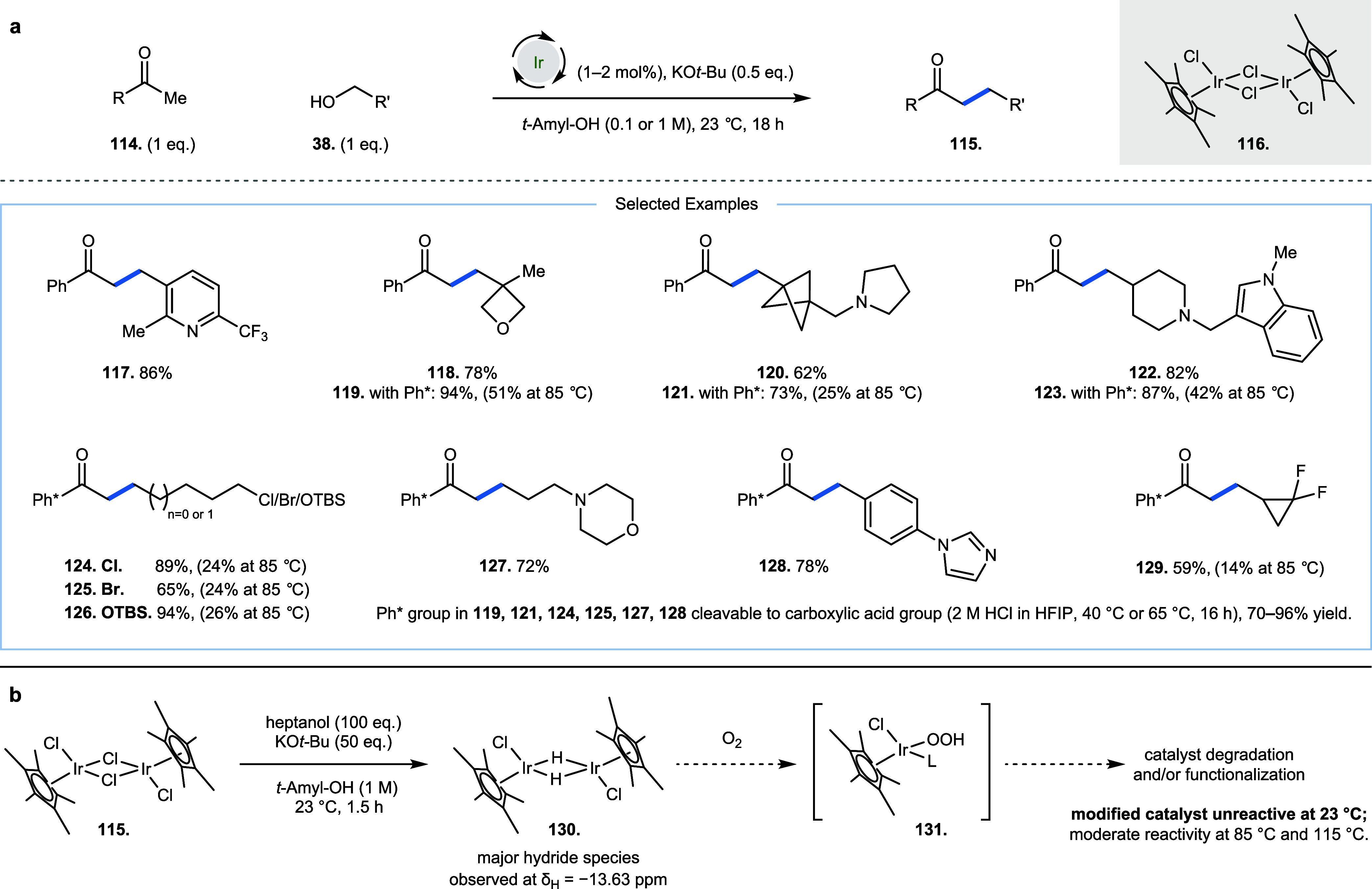
Room-temperature iridium-catalyzed alkylation of ketones with primary alcohols. (a) Functional group-tolerant ketone alkylation. (b) Importance of anaerobic conditions in this reaction at room temperature.

## 4 Room-Temperature C–N Bond-Forming Hydrogen Borrowing Reactions

In 2013, Andersson and co-workers reported a solvent-free iridium-catalyzed alkylation of anilines (**132**) with alcohols (**38**).^33^ Reactions were initially performed at 50 °C for 24 h; however, many of the substrates performed equally well at room temperature for 48 h (Figure 8a, **133**–**138**). This reaction required a bidentate iridium *N*-heterocyclic-carbene-phosphine complex **139**, which is synthesized in one step from [Ir­(cod)­Cl]_2_. Unactivated alcohols such as ethanol and 3-phenylpropan-1-ol were shown to give high yields of alkylated aniline products (**135** and **136**, 90% and 93% yield, respectively); this is in contrast to previous publications, which reported more moderate yields with nonbenzylic or nonallylic alcohols.^7,21,34^ In 2014, Enyong and co-workers disclosed a similar C–N bond-forming hydrogen borrowing reaction (Figure 8b).^35^ Again, initial work focused on higher temperature conditions (40–110 °C), but later, the authors showed that [Ru­(*p*-cymene)­Cl_2_]_2_ **143** precomplexed with a phenylalanine-derived ligand **142**, could enable these reactions at room temperature in 46–48 h (**144**–**148**). Up to 12 mol % ruthenium catalyst and 24 mol % ligand were used for some substrates at room temperature, and the alcohol is used as solvent (4 equiv when using ethanol). However, this paper displays improved diversity in the scope of amines used, and employs unactivated alcohols such as 1-butanol (**145**, **146**, **148**, all >99% conversion). An improved approach was described by Martín-Matute and co-workers in 2024 (Figure 8c).^36^ With 1.5 mol % iridium catalyst (**149**), anilines (**150**) could be coupled with benzylic alcohols (**151**) after either 15 h (**153**) or 36 h (**154**–**156**) in HFIP in high yield (**152**). The authors describe the beneficial effect of the NHC-amine ligand, which contrasts their own earlier work that had utilized a similar ligand incorporating an alcohol group instead of the amine.^37^ It was rationalized that the amino group is predominantly coordinated to the iridium center (unlike the alcohol group), which prevents formation of an intermolecular hydrogen bond from the lone pair of nitrogen to the hydrogen of the aniline substrate, preventing formation of an inactive resting state as observed with the former NHC-alcohol ligand. Notably, this reaction included 1:1 equivalence of the amine and alcohol reagents, which is an often-overlooked element of hydrogen borrowing alkylation reactions. Benzyl amines could also be coupled with alcohols; however, this required an elevated reaction temperature (**157**, 50 °C, 48 h). The limitations of this methodology were observed when using aliphatic amines.

**Figure 8:**
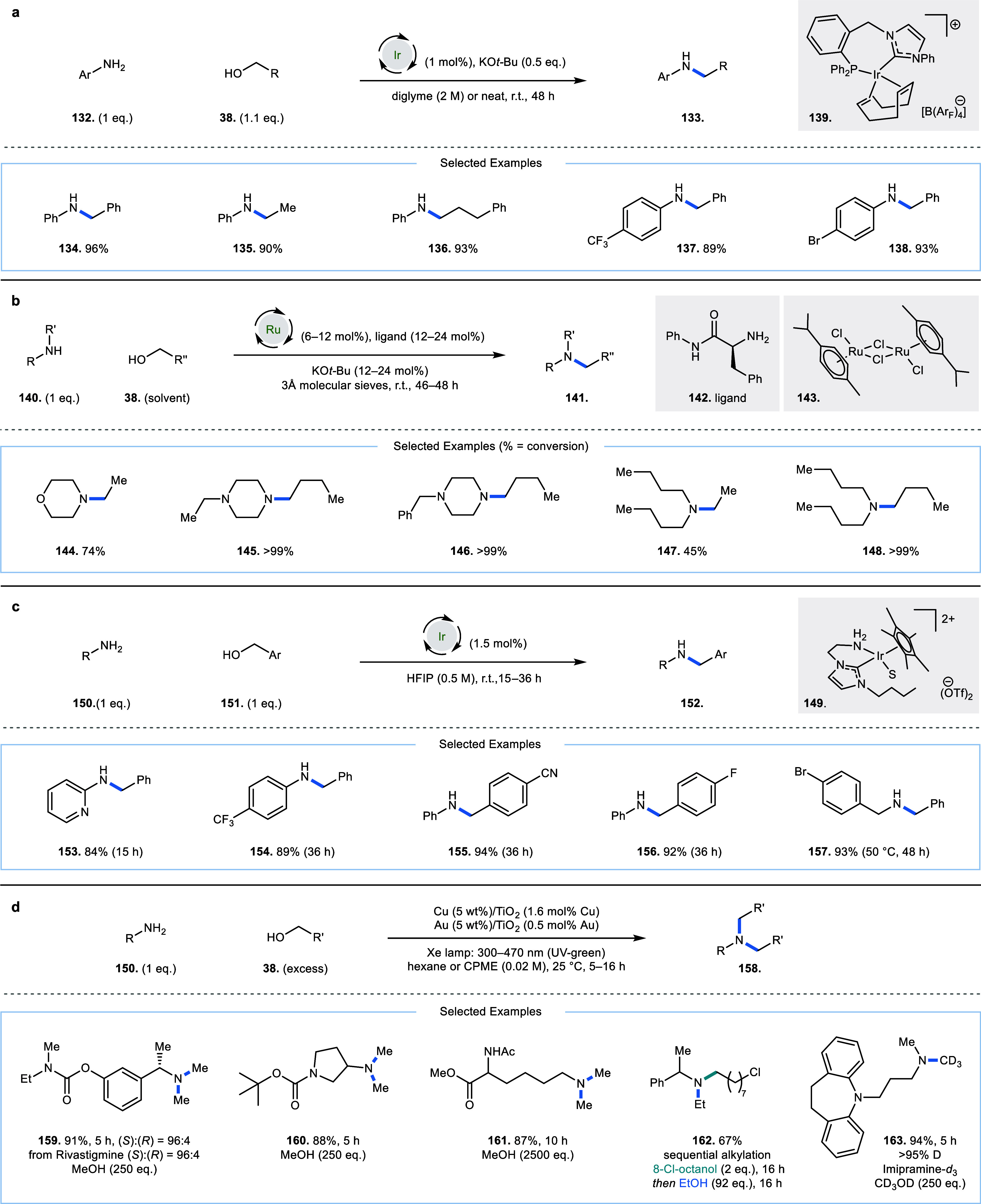
Room-temperature carbon–nitrogen bond-forming hydrogen borrowing alkylation reactions. (a) Iridium-catalyzed alkylation of anilines. (b) Ruthenium-catalyzed alkylation of amines with aliphatic alcohols. (c) Iridium-catalyzed alkylation of anilines in HFIP, S = solvent. (d) Functional group-tolerant visible light-driven alkylation of amines with aliphatic alcohols via heterogeneous catalysis.

Heterogeneous photocatalysts have also shown their utility in room temperature hydrogen borrowing reactions. Both organic benzimidazolium-based porphyrin^38^ and Cu–Au– or Cu–Mo-doped TiO_2_ can effect C–N bond-forming hydrogen borrowing reactions in the presence of UV light at low temperatures.^39,40^ Generally, these reactions proceed via excitation of electrons from the valence band to the conduction band, which enables oxidation of an alcohol and reduction of a condensation product, respectively (via single electrons with concomitant loss or gain of protons, respectively); however, metal hydrides have also been invoked as intermediates in some of these reactions.^40^

In 2018, Wheatley, Saito, Naka, and co-workers reported the room-temperature alkylation of pharmaceutically relevant amines (**150**) with a mixed heterogeneous catalyst prepared *in situ* from Cu/TiO_2_ and Au/TiO_2_, with activation by 300–470 nm light (broad source emitting a range from UV to visible green light) provided by a 300 W Xe lamp (Figure 8d, **158**–**163**).^40^ Temperature was maintained at 25 °C by a cooling circulator. The scope of amines used was diverse, including carbamate (**159**, **160**), ester (**161**), and secondary amide (**161**) functional groups and alcohols containing alkyl chloride groups were well tolerated (**162**). The authors also demonstrated the reaction with (*S*)-Rivastigmine (**159**), a drug used for Alzheimer’s treatment, which suffered no loss in enantioenrichment at the benzylic stereogenic center α to the amine group (96:4 e.r., for both starting material and product). Substrates containing tetra-substituted alkene and pyridine groups required elevated temperatures (50 °C), and secondary alcohol functional groups were notably unaffected at this elevated temperature. While other similar heterogeneous room temperature approaches have been published,^38,39^ this paper is notable for its exemplification of drug-like amines. The requirement for UV radiation may limit functional group tolerance somewhat, since higher-energy light can directly excite some functional groups, encouraging side reactions, and using a light source emitting a broad range of wavelengths in the UV range can exacerbate this issue.^41^ UV light also creates practical barriers to utility since the reactions have to be fully contained, to limit exposure of the radiation to users.^42,43^ While the focus of this review is on homogeneous catalysis, this report of heterogeneous catalysis was noted for being able to manipulate complex molecules at room temperature.

In 2020, Wang and co-workers (Figure 9a)^44^ and Jin, Xing, and co-workers (Figure 9b)^45^ reported the enantioselective synthesis of secondary alcohols bearing β-amino groups. In the presence of a chiral ruthenium catalyst **56** or **64**, racemic allylic alcohols **53** can be oxidized to ketones and undergo a conjugate addition by amine **140**, followed by enantioselective carbonyl reduction to liberate secondary alcohol products **164** and **170**, respectively. Wang and co-workers employed catalyst **56** (1–2 mol %) and K_3_PO_4_ (1.5 equiv) in toluene at 30 °C (**165**–**169**). The work of Jin, Xing, and co-workers is notable for using catalyst **64** at a particularly low loading (0.25 mol %), and with catalytic amounts of KO*t*-Bu base (15 mol %, **170**–**174**). While enantioselective reduction of carbonyl compounds by chiral ruthenium complexes is well precedented,^46–53^ and has recently been reported at room temperature,^21,54^ these reactions are welcomed for their exemplification of a highly diverse scope of amines. This includes the use of Cytisine (**168**) and Amoxapine (**169**) demonstrating the viability of pyridone and amidine functionality, respectively. In addition, pyridine (**167**), pyrimidine (**171**), thiomorpholine (**173**), piperazine (**167**, **169**, **171**, **172**, **174**), quinoline, benzofuran (**173**), thioether, amide (**172**), and Boc groups. Both alkyl amines and anilines were effective, delivering products with high enantioselectivity. The regioselectivity of the intermediate reaction, as well as the diverse scope of these reactions (110 examples total), exemplifies the benefits of room temperature hydrogen borrowing. Similar reactivity has been described with an earth-abundant manganese catalyst Mn­(CO)_5_Br, together with a chiral macrocyclic amine ligand at 40 °C with a narrower scope.^55^

**Figure 9:**
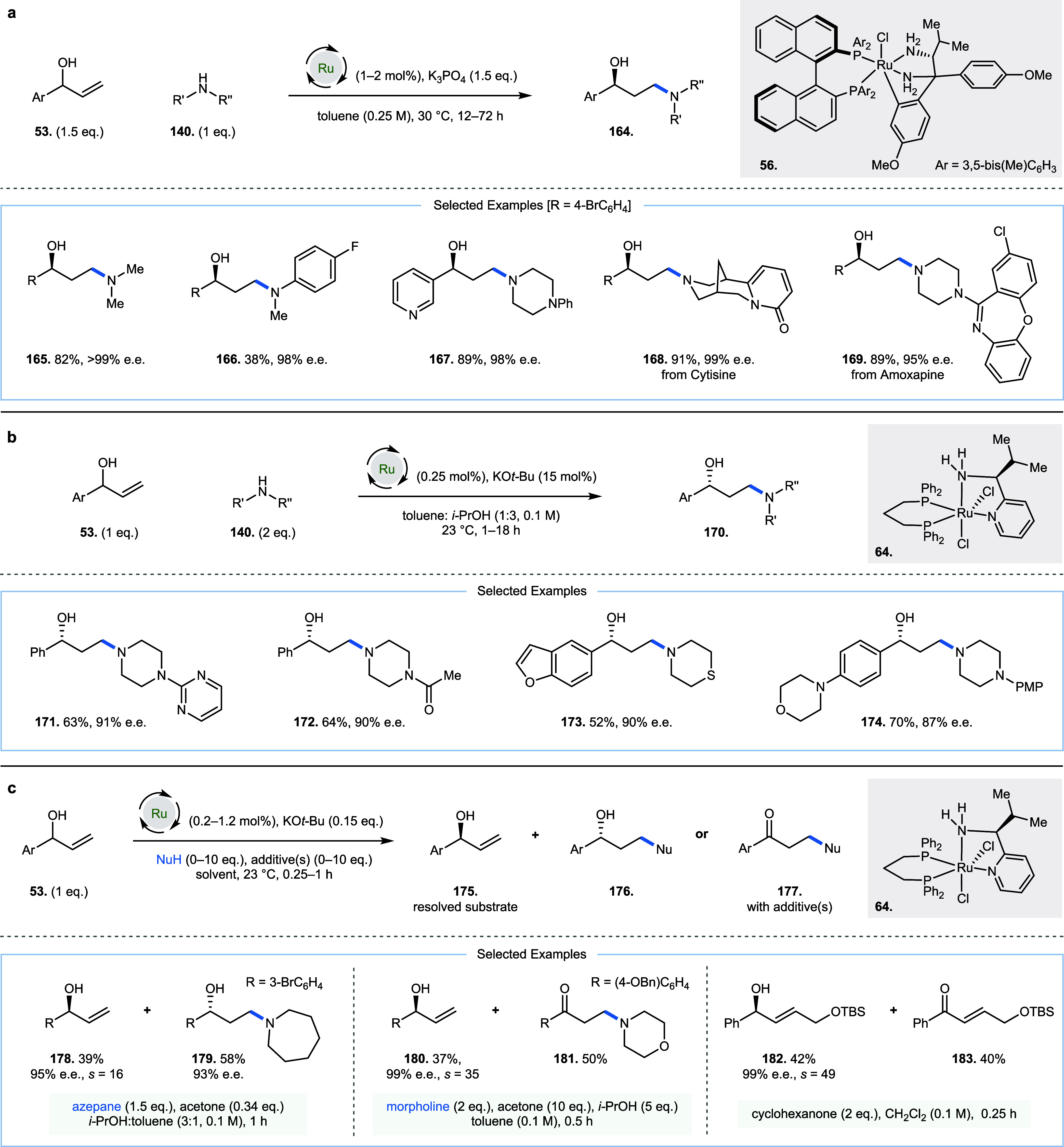
Enantioselective carbon–nitrogen bond-forming hydrogen borrowing alkylation of allylic alcohols. (a, b) Ruthenium-catalyzed alkylation with amines, Ar = 3,5-bis­(Me)­C_6_H_3_. (c) Kinetic resolution of allylic alcohols, where *s* is the selectivity factor.

Finally, in related work in 2022, Yu, Xing, and co-workers demonstrated the kinetic resolution of allylic alcohols with chiral diphosphine, diamino ruthenium catalyst **64** at room temperature with a variety of related reaction conditions (Figure 9c, **175**–**183**).^56^ Racemic allylic alcohols **53** could be resolved to generate enantioenriched alcohols **175** (via oxidation and enantioselective reduction) as well as γ-functionalized alcohols (**176**, via oxidation, nucleophilic addition, and alternate enantioselective reduction) or β-functionalized ketones (**177**, via oxidation and nucleophilic addition) at room temperature. β-Functionalized ketone formation was promoted by the addition of a hydride acceptor such as acetone (see **178** and **179**) and substituted allylic alcohol substrates could be resolved without an intermediate nucleophilic addition reaction (**182** and **183**) using cyclohexanone as a hydride acceptor. DFT was used to unveil the role of π-π interactions between: (i) the aryl substituent at the *in situ* generated ketone or α,β-unsaturated ketone (e.g., **181** or **183**) and (ii) the phenyl group of the phosphine ligand of the catalyst (shown in **64**) in the transition state for enantioselective ketone reduction.

## 5 Conclusion and Outlook

Hydrogen borrowing is an attractive strategy in comparison to traditional alkylation reactions with alkyl halides: alcohols are stable, commercially abundant, and safe to handle, the method can be catalytic in base, and water is generated as the sole byproduct. However, many of these reactions depend on high operating temperatures in the presence of strong bases to proceed (76–200 °C). In the past decade, a handful of metal-catalyzed room temperature hydrogen borrowing reactions have emerged that demonstrate broader functional group tolerance as well as (regio- and enantio-)­selective alkylation. Reaction conditions in early publications were typically liberal with respect to long reaction times, use of excess alcohol reagents, and high catalyst loading, but crucially demonstrated that the catalytic regime was viable at room temperature. More recently, the exemplification of complex molecules, containing sensitive or reactive functional groups, nitrogen-rich heterocycles, or strained rings, has been realized at room temperature; all while using low catalyst loading and reactants in stoichiometric unity. More than half of these reactions are enantioselective, which includes both C–C and C–N bond-forming reactions, and regioselective alkylation (between primary and secondary alcohol, for example) can be enabled at room temperature as nonproductive intermediate reactions are disfavored at this lower temperature.

One natural limitation of these methods is that the intermediate (condensation) reaction must be productive at room temperature. Future approaches to enabling an intermediate reaction at room temperature need to be tolerant of the metal-catalyzed elementary steps, and enamine catalysis has proven useful, catalytically enabling previously nonfunctioning intermediate reactions and simultaneously doing so (diastereo- or enantio-)­selectively (via proline-derived catalysts). We would welcome a systematic investigation into substrate and catalyst classes that are productive in the individual elementary steps of hydrogen borrowing, particularly oxidation, a selection of intermediate reactions, and reduction at room temperature. This could derive which hydrogen borrowing reactions (e.g., between chosen substrates) can operate at a particular temperature with currently outlined strategies, but then most importantly, after elucidating the challenging step(s), help focus attention to apply or devise new strategies to overcome this limitation.

We have also observed that metal-binding heteroatom-containing substrates required moderately higher temperatures to be rendered tolerant in hydrogen borrowing—due to metal complexation/sequestration, inhibiting the oxidation step by preventing effective β-hydride elimination. An alternate approach to overcome this could be combining transition metal catalysis with photochemistry: a metal catalyst capable of entering an excited state and performing the elementary steps of hydrogen borrowing, namely, ligand exchange, oxidation, or reduction—could render these steps rate-enhanced in the excited state. Beyond this, mechanistically distinct methods such as biocatalysis, which achieves hydrogen borrowing via NADP^+^ and NADPH cofactors (and notably not via a catalytic metal), could be useful. We also recognize that particular functional groups—for example, an *aryl* ketone or a *benzylic* alcohol, can sometimes be required to demonstrate hydrogen borrowing on some substrates. This calls for the development of improved and selective catalysts. The reactions discussed were accomplished with a diverse range of metal catalysts (including Fe, Ru, Ir, and Cu); the success of earth-abundant metal catalysts in these reactions is particularly encouraging.

It is our concluding view that (i) the derivation of key reactivity relationships between substrate and catalyst classes in the elementary steps of hydrogen borrowing; (ii) the discovery of superior earth-abundant metal catalysts; and (iii) the application of these catalysts to hydrogen borrowing reactions involving a broader set of intermediate (condensation) reactions would help to develop the field as a whole. The progression thus far augurs well for the development of sustainable, functional group diverse, and selective hydrogen borrowing reactions that we envisage can find application across discovery and process chemistry.
